# New records of *Caribbomerus* from Hispaniola and Dominica with redescription of *C. elongatus* (Fisher) and a key to species of the genus in the West Indies (Coleoptera, Cerambycidae, Cerambycinae, Graciliini)

**DOI:** 10.3897/zookeys.85.862

**Published:** 2011-03-11

**Authors:** Steven W. Lingafelter

**Affiliations:** Systematic Entomology Laboratory, Plant Sciences Institute, Agriculture Research Service, U.S. Department of Agriculture, National Museum of Natural History, Washington, D.C. 20013-7012, U.S.A.

**Keywords:** Dominican Republic, Haiti, longhorned woodboring beetles, taxonomy, faunistics

## Abstract

Three species of *Caribbomerus* Vitali are newly recorded for the Dominican Republic: *Caribbomerus decoratus* (Zayas), *Caribbomerus elongatus* (Fisher), and *Caribbomerus asperatus* (Fisher). The first two also represent first records for Hispaniola. *Caribbomerus elongatus* (Fisher) is redescribed based on additional material, including the first known males. *Caribbomerus similis* (Fisher) is newly recorded for Dominica. A key to the species of the genus from the West Indies is provided.

## Introduction

Only one genus of Graciliini occurs in the West Indies, *Caribbomerus* Vitali (formerly known under the preoccupied *Merostenus* Walker and formerly placed in the Callidiopini) (Vitali and Rezbanyai-Reser 2003; Monné and Bezark 2010). *Caribbomerus* is most easily recognized by the following combination of characters: long, narrow body (4–11 mm long; 0.7–1.9 mm wide); elongate, usually parallel-sided elytra (elytron length 5–9 times its width); anterior coxal cavities closed posteriorly by a procoxal process which is extremely narrow between the procoxae and then abruptly widened behind; coarsely faceted eyes; and antennae distinctly longer than body (in part, based on Martins and Galileo 2005). Other characters include: third antennomere distinctly shorter than all others (with exception of pedicel); elytral apex of most species impunctate and with integument smooth and distinctly paler than remainder; prothorax 0.16–0.25 length of body and widest at or near posterior third in most species.

Prior to Napp and Martins (1984), *Caribbomerus* was known only from the Antilles. With the species they described from Brazil, Mexico, and Jamaica, along with a species Micheli (2003) described from Puerto Rico, the number of species was increased to twelve.

**Figure 1.**
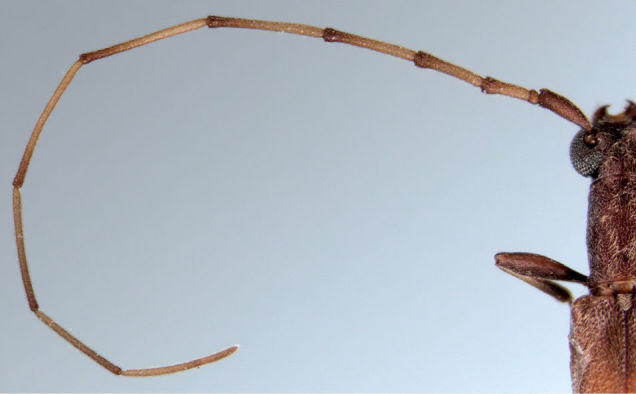
Habitus montage images of *Caribbomerus elongatus* (Fisher), male.** a** Dorsal **b** ventral.

**Figure 2. F2:**
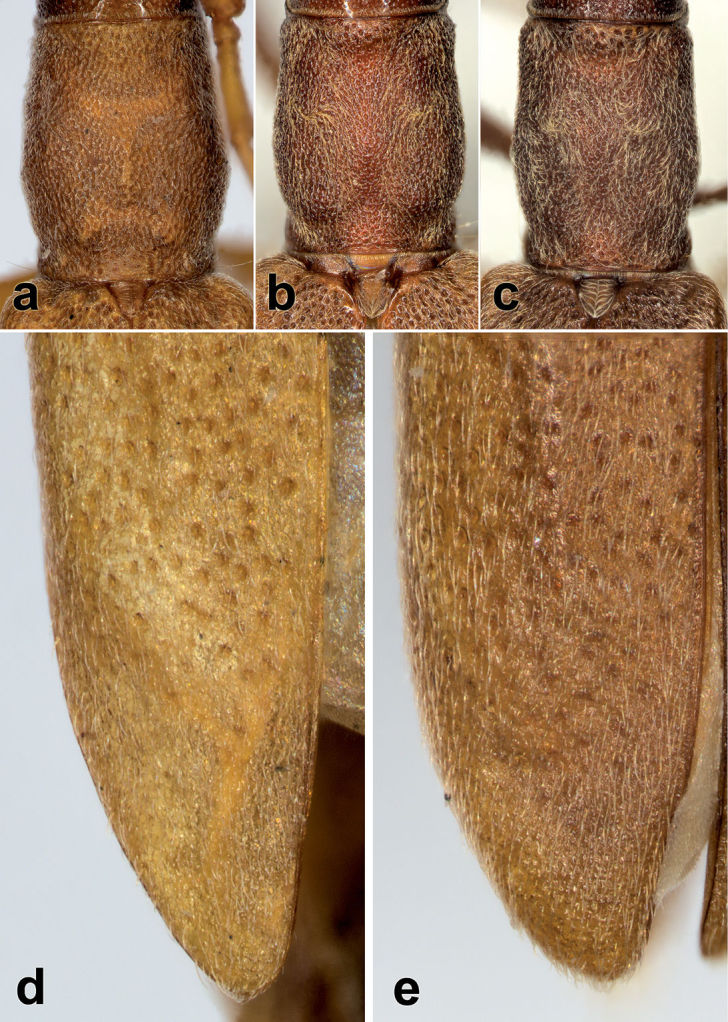
Antenna of *Caribbomerus elongatus* (Fisher) male.

**Figure 3. F3:**
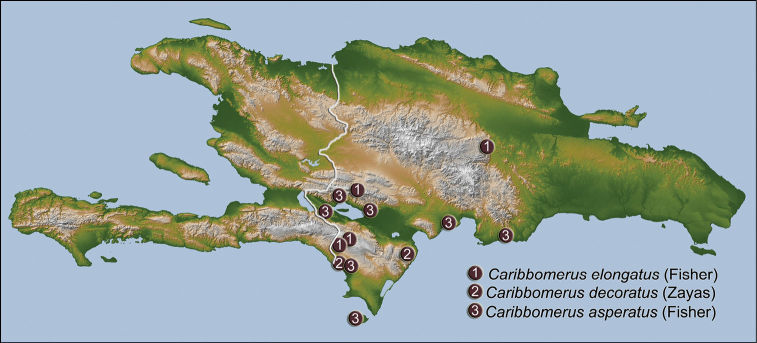
*Caribbomerus elongatus* (Fisher) male. **a** Scutellum **b** Prosternum and mesosternum **c** head **d** apex of abdomen showing parameres.

**Figure 4.**
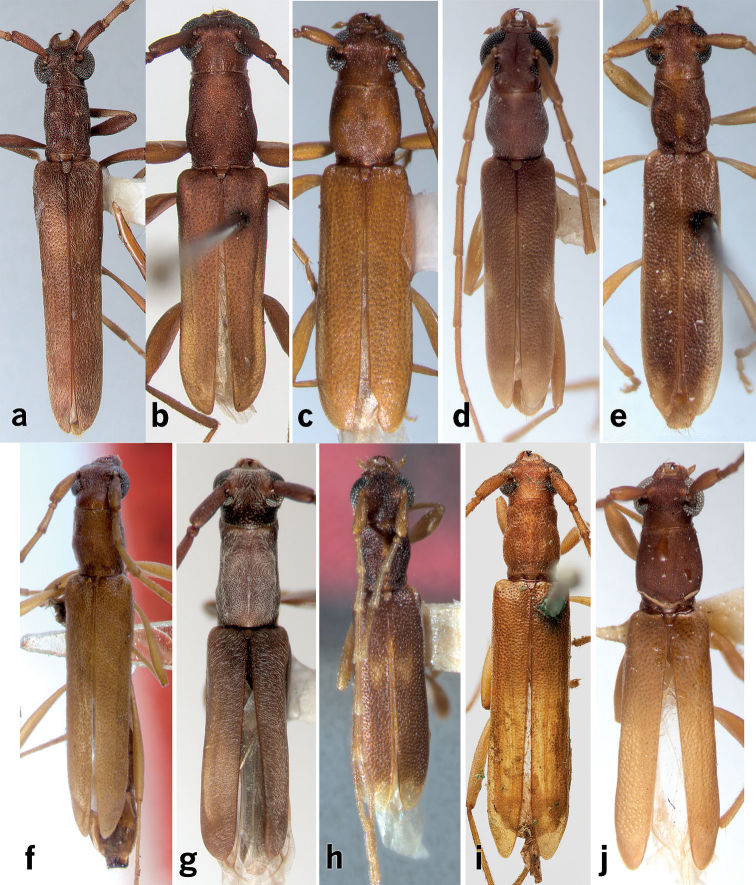
Pronotum. **a** *Caribbomerus elongatus* (Fisher) holotype female **b** *Caribbomerus elongatus* (Fisher) female **c** *Caribbomerus elongatus* (Fisher) male. Elytral apex. **d** *Caribbomerus elongatus* (Fisher) holotype female **e** *Caribbomerus elongatus* (Fisher) male.

**Figure 5.**
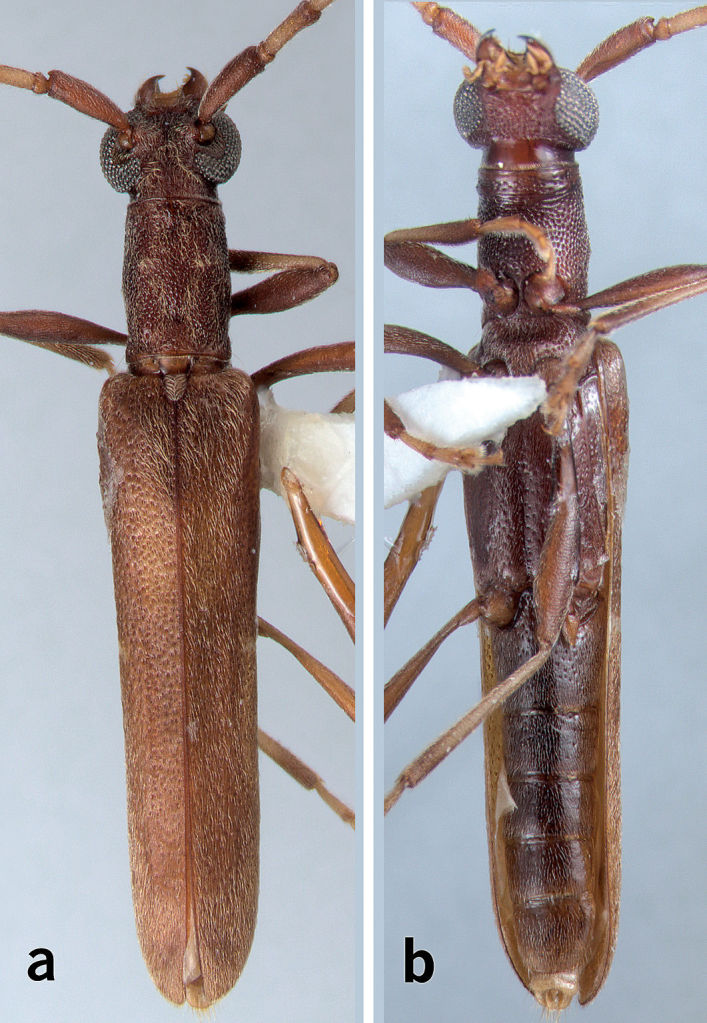
Dorsal habitus photographs of West Indian *Caribbomerus* species. **a** *Caribbomerus elongatus* (Fisher) male **b** *Caribbomerus asperatus* (Fisher) **c** *Caribbomerus attenuatus* (Chevrolat) **d** C. charynae (Micheli) **e** *Caribbomerus decoratus* (Zayas) **f** *Caribbomerus elongatus* (Fisher) holotype female **g** *Caribbomerus exiguus* (Zayas) **h** *Caribbomerus picturatus* (Napp and Martins) **i** *Caribbomerus productus* (White) **j** *Caribbomerus similis* (Fisher).

**Figure 6.**
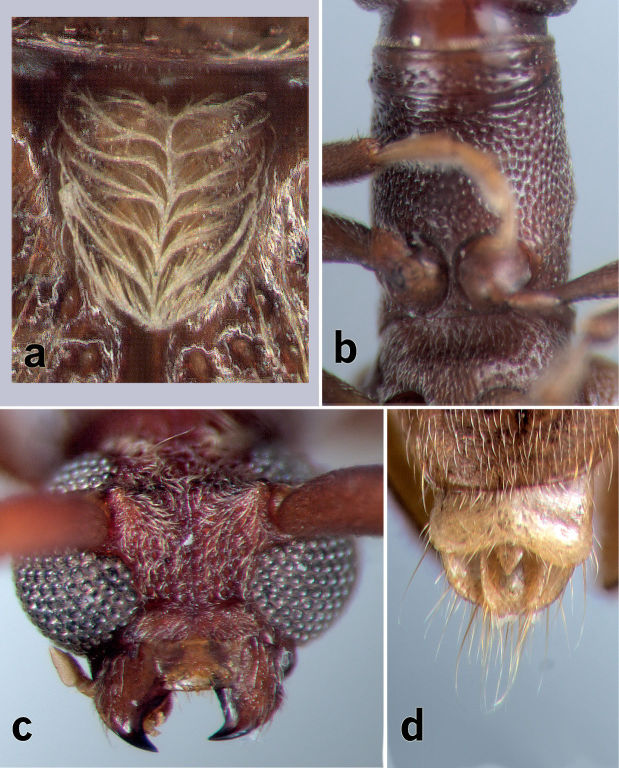
Distribution map for the three *Caribbomerus* species known from Hispaniola.

Three species of *Caribbomerus*: *Caribbomerus decoratus* (Zayas), *Caribbomerus elongatus* (Fisher), and *Caribbomerus asperatus* (Fisher) are recorded from the Dominican Republic (new country records) (unrecorded in Monné and Bezark 2010; Perez-Gelabert 2008). For the first two, Hispaniola represents a new island record. *Caribbomerus elongatus* is redescribed based on additional material (including newly discovered males) revealing broader morphological variation than reflected in the original description. All Hispaniolan *Caribbomerus* are restricted to the southern and southwestern parts of the Dominican Republic, along and southwest of the Cordillera Central (Fig 6). A key, table of measurements (Table 1), and photos (Figs 1–5) to all the species of *Caribbomerus* from the West Indies are provided.

## Methods

Most of the material examined in this study was collected by R. H. Turnbow, Jr. (RHTC), E. F. Giesbert (FSCA), J. Rawlins and R. Davidson (CMNH), and M. Ivie and K. Guerrero (WIBF). Holotypes are deposited in the Smithsonian Institution (USNM) and images are available in the online Smithsonian Primary Type database (Lingafelter et al. 2010).

Images were captured with a Zeiss AxioCam HRc camera mounted on a Zeiss Discovery V20 stereomicroscope with Sycop motorized zoom and focus control. Objectives used included a PlanApo S 1.0× and 0.63×. For illumination, a Zeiss KL 2500 LCD with ring light attachment was used. Axiovision software enabled preparation of montaged images and precise, automatically calibrated measurements.

In the course of this work, these collections and others were examined for *Caribbomerus* specimens. Nearns et al. (2006) was consulted for species in the Fernando Zayas collection as well as the online type image databases of the Museum of Comparative Zoology, Harvard University (MCZC 2010), and the American Museum of Natural History (AMNH 2010). The acronyms and contact persons are listed below:

BMNH The Natural History Museum, London, England (Sharon Shute, Max Barclay)


CMNH Carnegie Museum of Natural History, Pittsburgh, Pennsylvania, U.S.A. (J. Rawlins, R. Davidson, R. Androw)


CNCI The Canadian National Collection of Insects, Ottawa, Ontario, Canada (S. Laplante, O. Lonsdale)


EFGC Edmund F. Giesbert Collection (at FSCA), Gainesville, Florida, U.S.A. (M. Thomas, P. Skelley)


FSCA Florida State Collection of Arthropods, Gainesville, Florida, U.S.A. (M. Thomas, P. Skelley)


FSPC Frederick W. Skillman Private Collection, Pearce, Arizona, U.S.A. (F. W. Skillman)


RHTC Robert H. Turnbow, Jr. Private Collection, Ft. Rucker, Alabama, U.S.A. (R. H. Turnbow)


USNM National Museum of Natural History, Smithsonian Institution, Washington, DC, U.S.A. (S. Lingafelter)


WIBF West Indian Beetle Fauna Project, Bozeman, Montana, U.S.A. (M. Ivie)


### Caribbomerus elongatus

(Fisher)

Figs 1 [Fig F2] [Fig F3] 4 5a,f 6 Table 1

#### Diagnosis.

Based on the availability of additional non-type material (including males), the variation within this species can now be more fully documented. Although the holotype female has uniformly pale testaceous antennae, the antennae of males are narrowly dark annulate at the apices of most antennomeres (except scape and pedicel). The pronotum of females is slightly swollen posteriorly and then moderately constricted at the base but in males it is nearly parallel-sided and not or barely wider at posterior third unlike most of the remaining species of the genus. The pronotum of both sexes is densely punctate throughout, but in some females there is a very small, vaguely defined median callus lacking punctures and surrounded by a slight depression. The pronotum and elytra have a coating of tawny, appressed pubescence which does not obscure punctures. The elytral apices are abruptly smooth and impunctate with the anterior margin of this region slightly depressed.

#### Redescription.

Small size, 6.63–7.99 mm long; 1.19–1.82 mm broad; integument reddish-brown, with pronotum, head, and base of elytra slightly darker. Head with sparse, tawny pubescence not obscuring punctation; sparsely but distinctly punctate. Eye lobe coarsely faceted; completely, widely separated; strongly protuberant laterally (nearly projecting as wide as humeri;) lower lobe occupying nearly all of head from lateral view; upper lobe much smaller; lobes connected by 5 facets at narrowest point; broadly separated by two-thirds width of pronotum on vertex behind antennal tubercles. Interantennal impression very deep with antennal tubercles strongly elevated in V-shape. Antennae of males extending approximately 1.5× length of body in males, 1.3× length of body in females; covered with fine, inconspicuous, short, translucent pubescence. Antennomeres 3–11 of males noticeably dark brown at extreme apices, otherwise, pale brown (antennomeres uniformly pale testaceous in females). Scape extending beyond anterior pronotal margin; integument smooth, not asperate. Other than antennomere 2, antennomere 3 shortest; remaining antennomeres successively increasing in length to 9; 9–11 subequal. Antennomere lengths as follows: scape: 0.59–0.95 mm; antennomere 3: 0.42–0.69 mm; antennomere 4: 0.75–1.14 mm; antennomere 5: 0.95–1.48 mm; antennomere 6: 1.21–1.86 mm. Pronotum distinctly longer than broad, cylindrical in male; distinctly narrower than elytral base (in female, less cylindrical, slightly swollen at posterior third); sparsely, tawny pubescent; distinctly punctate, but punctures small and non-contiguous; without distinct calli, but with small anterior and posterior depressions in male (female with small, vague middle callus surrounded by depression on pronotal disk). Pronotal length: 1.19–1.57 mm; pronotal width: 0.78–1.13 mm; pronotal length/width: 1.3–1.6. Pronotum about 0.18–0.19× length of body. Prosternum sparsely pubescent; densely, closely punctate. Elytron pale reddish brown, slightly darker at base (with vague, darker areas at humerus, middle, and apical third in a few specimens); distinctly but separately punctate; punctures becoming slightly sparser posteriorly and then abruptly disappearing from apices; anterior margin of impunctate apices slightly depressed; sparsely tawny pubescent (pubescence becoming denser at apices); very long and narrow, narrowly rounded at apices. Elytron length: 4.62–5.49 mm; elytron width: 0.49–0.76 mm; elytral length/width: 7.2–9.4. Scutellum sparsely tawny pubescent; setae joining together and at middle; rounded posteriorly; not noticeably punctate. Hind Leg moderately long, but femur not attaining abdominal apex; reddish brown; sparsely covered in short pubescence, only weakly expanded apically. Tibiae (particularly metatibia) narrow, elongate, straight, somewhat darkened apically. Venter sparsely pubescent, darker than dorsum. Abdomen of both sexes with margin of fifth sternite evenly, broadly rounded.

**Table 1.** Anatomical measurements (in millimeters) and proportions in West Indian *Caribbomerus* species. **BL** = body length; **EL** = elytron length; **EW** = elytron width; **PL** = pronotum length; **PW** = pronotum width.

Taxon	BL	EL	EW	PL	PW	BL/PL	EL/EW	PL/PW
Caribbomerus asperatus	4.27–6.53	2.50–3.73	0.46–0.78	0.94–1.61	0.78–1.32	4.5–5.6	4.8–5.4	1.2
Caribbomerus attenuatus	4.08–5.381	2.67–3.52	0.46–0.56	0.84–1.26	0.74–0.90	4.3–4.9	5.8–6.3	1.1–1.4
Caribbomerus charynae	5.81	3.71	0.53	1.05	0.88	5.53	7.0	1.1
Caribbomerus decoratus	5.112–5.68	3.47–3.79	0.50–0.52	0.98–1.22	0.73–0.80	4.6–5.2	6.9–7.3	1.3–1.5
Caribbomerus elongatus	6.633–7.99	4.62–5.49	0.49–0.76	1.19–1.57	0.78–1.13	5.1–5.6	7.2–9.4	1.3–1.6
Caribbomerus exiguus	5.614	3.36	0.56	1.32	0.81	4.2	6.0	1.6
Caribbomerus picturatus	4.4–4.5	2.8–2.9	0.40–0.45	1.00	0.60–0.65	4.4	6.4–7.0	1.6
Caribbomerus productus	4.5–10.0	6.92	0.86	1.88	1.42	5.3	8.0	1.3
Caribbomerus similis	5.925	3.78	0.54	1.18	0.99	5.0	7.0	1.1

1 Zayas (1975) reports on specimens from Cuba as large as 6.5 mm (outside the range I have seen). 2 Zayas (1975) reports the holotype as 4.5 mm (outside the range I have seen from Hispaniola). 3 Zayas (1975) reports a specimen as small as 5.5 mm (outside the range I have seen from Hispaniola). 4 Zayas (1975) reports a specimen as small as 4.0 mm.5 Chalumeau and Touroult (2005) reports this species as large as 6.8 mm.

#### Discussion.

Using the key to *Caribbomerus* of Vitali and Rezbanyai-Reser (2003), this species would run nearest *Caribbomerus howdeni* (Napp and Martins), a Mexican species, based on the coloration, and shape and pubescence of the pronotum. However, their key is incorrect in coding *Caribbomerus elongatus* as having a glabrous pronotum. The holotype of *Caribbomerus elongatus* (and all of the new material seen) clearly has translucent pubescence on the pronotum as noted in Fisher’s (1932) description. This species, previously known by the female holotype from Cuba (Fisher 1932; Monné and Bezark 2010) and an unspecified number from Oriente, Las Villas, and Pinar del Rio provinces in Cuba as mentioned by Zayas (1975), is now documented for the Dominican Republic (new island & country record).

#### Specimens.

Holotype (female): Cuba, Wajay, Havana, 15 December, 1930, S. C. Bruner, coll. (USNM). Dominican Republic: San Juan Prov., Sierra de Neiba, trail to Sabana de Silencio, 10 km SSW of El Cercado, 1650-1700 m, 10-11 July, 2006, A. Konstantinov, coll. 18°39.935'N, 71°31.964'W; 18°39.935'N, 71°31.964'W (1 male, USNM); Dominican Republic: Independencia Prov. P. N. Sierra de Bahoruco around Caseta no. 1, 18°16.038'N, 71°32.691'W, December 11-12, 2003, D. Perez, R. Bastardo, B. Hierro, RD#191 (1 male, USNM); Dominican Republic: Independencia, Sierra de Bahoruco, north slope, 13.5 km SE Puerto Escondido, 18°12.24'N, 71°30.54'W, 1807 m, 24-26 Mar 2004, R. Davidson, J. Rawlins, C. Young, C. Nuñez, M. Rial, broadleaf *Pinus* dense woodland, hand collected, Sample 41243 (1 male, CMNH); Dominican Republic: Independencia, Sierra de Bahoruco, Lomo del Toro, 18°17.16'N, 71°42.46'W, 2310 m, 7-8 November 2002, W. A. Zanol, C. W. Young, C. Staresinic, J. Rawlins, meadow in pine woods, hand collected, Sample 40149 (1 female, CMNH); Dominican Republic: Pedernales, 5 km NE Los Arroyos, 18°15'N, 71°45'W, 1680 m, 17-18 July 1990, J. E. Rawlins, S. Thompson (1 female, CMNH); Dominican Republic: La Vega Prov., Cordillera Central, Loma Casabito, 15.8 km NW Bonao, 19°02.12'N, 70°31.08'W, 1455 m, 28 May 2003, J. Rawlins, C. Young, R. Davidson, C. Nunez, P. Acevedo, evergreen cloud forest, east slope, UV light, sample 21312 (3 females, CMNH).

### Caribbomerus decoratus

(Zayas)

Figs 5e 6 Table 1

#### Diagnosis.

This species is recognized by the densely, coarsely punctate pronotum with a distinct (and sometimes paler), mostly impunctate median longitudinal callus surrounded by less distinct and mostly punctate peripheral calli (one on either side). *Caribbomerus decoratus* (Zayas) is very similar to *Caribbomerus picturatus* (Napp and Martins) which is known only from Jamaica. In both species, the elytra have a vague, pale macula at the middle near the suture and have the elytral apices impunctate and pale. The leg color of *Caribbomerus decoratus* ranges from uniformly pale yellow to reddish-brown with the clavate portions of the femora darker than the rest of the legs (in *Caribbomerus picturatus* (Napp and Martins) the legs are uniformly pale yellow and much paler than the dorsum). *Caribbomerus picturatus* also can be distinguished from *Caribbomerus decoratus* by the heavy surface sculpture and contiguous punctation of the frons, vertex, and antennal tubercles. In *Caribbomerus decoratus*, the punctures are sparse and not contiguous.

#### Discussion.

This species was described by Zayas (1975) from Cuba. His description of the pronotum and exposed terminal tergites, among many other features, suggests that the photograph of this species is mislabeled in Nearns et al. (2006). In that paper, figure 5a should be *Caribbomerus exiguus* (Zayas) and figure 5b should be *Caribbomerus decoratus* (Zayas) (E. Nearns, pers. comm). This species, previously known only from Cuba (Zayas 1975; Monné and Bezark 2010), is now documented for the Dominican Republic (new island & country record).

#### Specimens.

Dominican Republic: Prov. Barahona, nr Filipinas, Larimar Mine 26 June – 7 July 1992, Skillman, Skelley, Woodruff, blacklight (FSPC, donated to USNM); Dominican Republic: Barahona, vic. Filipinas, 1700’, May 5-6 1985, E. Giesbert, coll (EFGC [FSCA]); Dominican Republic: Barahona, 2 km E. Payaso, 460 m, mv + bl, 13 July 1996, R. Turnbow (RHTC); Dominican Republic: Pedernales, Sierra Baoruco, 19 May 1992, R. Turnbow (RHTC).

### Caribbomerus asperatus

(Fisher)

Figs 5b 6 Table 1

#### Diagnosis.

Like *Caribbomerusribbomerus attenuatus* (Chevrolat) and *Caribbomerus similis* (Fisher), *Caribbomerus asperatus* has a very diagnostic rugulose pronotum that lacks calli and pubescence. It is recognized by a combination of features that include the matte finish on the elytra, micropunctation between elytral punctures, relatively uniform reddish-brown coloration above, distinctly elevated antennal tubercles, and elytral apices not distinctly impunctate.

#### Discussion.

This is the most common species of *Caribbomerus* in Hispaniola. It was originally described based on specimens from Haiti, and has not been previously recorded from the Dominican Republic (Fisher 1932, Monné and Bezark 2010). With numerous collection records listed below, this species is now documented for the Dominican Republic (new country record).

#### Specimens.

Dominican Republic: Pedernales Prov., 12 km N of Cabo Rojo, 18°03'N, 71°38'W, 250-350 m, blacklight, 9 July 2004, Daniel Perez / Steve Lingafelter (5, USNM); Dominican Republic: Independencia Prov., 3 km up road from La Descubierta to Los Pinos, 15 July 2004, blacklighting, S. W. Lingafelter (4, USNM); Dominican Republic: Azua Prov., entrance to Boca Vieja Marina near Biyeya Beach, 23.iv.2004, 19°25'N, 69°51'W, D. Perez, B. Hierro (night) RD#241 (3, USNM); Dominican Republic: Pedernales Prov., Cabo Rojo, Alcoa (EFGC [FSCA]); Dominican Republic: Pedernales Prov., 10.2 km N Cabo Rojo (EFGC [FSCA]); Dominican Republic: Bahoruco, 5.8 km SW Neiba, eastern playa of Lago Enriquillo, 3 April 2004, collector: J. Rawlins, R. Davidson, C. Young (CMNH); Dominican Republic: Isla Beata, near Pedernales, October (MCZC); Dominican Republic: Bani, August (MCZC); Dominican Republic: Pedernales Prov., Cabo Rojo, sea level, 22 August 1988, beating veg., M. A. Ivie, T. K. Philips, & K. A. Johnson colrs (WIBF); Dominican Republic: Independencia Prov., ESE Jimani, La Florida 18°24'N, 71°4'W, 20 m, at uv light 14 April 1993, M. A. Ivie, D. Sikes, W. Lanier (WIBF); Dominican Republic: Pedernales, 9.5 km N. Cabo Rojo, 33 m, 18°00.042'N, 71°38.793'W, 08 August 1999, lights and beating, M. A. Ivie, and K. A. Guerrero (WIBF).

### Caribbomerus similis

(Fisher)

Figs 5j Table 1

#### Diagnosis.

Like *Caribbomerus attenuatus* (Chevrolat) and *Caribbomerus asperatus* (Fisher), *Caribbomerus similis* (Fisher), has a rugulose pronotum that lacks calli and pubescence. It is distinguished from *Caribbomerus asperatus* by having the integument mostly light reddish-brown in color with the elytra distinctly paler than the pronotum, having the antennal tubercles unelevated, and in having the elytral apices distinctly impunctate. It is distinguished from *Caribbomerus attenuatus* in having a matte integument with micropunctation between punctures.

#### Discussion.

This is an uncommonly collected species. It was originally described from two specimens from Antigua (Fisher 1932). Monné and Bezark (2010) record it from Barbuda. One specimen was examined in the USNM from Dominica and this is a **new country record**.

#### Specimens.

Dominica: Grande Savane, February 3, 1965, J. F., G. C., and T. M. Clarke (USNM).

## Key to *Caribbomerus* of the West Indies

**Table:** 

1	Pronotum with impunctate, non-rugulose, or non-asperate median callus	2
–	Pronotum without impunctate, non-rugulose, or non-asperate median callus	4
2(1)	Elytra with pale spot near suture at middle	3
–	Elytra nearly uniformly colored, without pale spot at suture near middle. Cuba, Hispaniola	*Caribbomerus elongatus* (Figs 1–4)
3(2)	Frons, vertex, and antennal tubercles with dense, contiguous punctation. Jamaica	*Caribbomerus picturatus* (Fig. 5h)
–	Frons, vertex, and antennal tubercles with sparse punctation. Cuba, Hispaniola	*Caribbomerus decoratus* (Fig. 5e)
4(1)	Pronotum with distinct punctures	5
–	Pronotum without distinct punctures (either asperate or rugulose)	6
5(4)	Pronotum with dense, contiguous punctures throughout. Scutellum with distinct pubescence joining together at sides, apex, and middle as in Fig. 3a. Cuba, Hispaniola	*Caribbomerus elongatus* (Figs 1–4)
–	Pronotum with punctures mostly separated by at least their diameter. Scutellum glabrous or with reduced pubescence (not as in Fig. 3a). Jamaica	*Caribbomerus productus* (Fig. 5i)
6(4)	Pronotum coated with sparse, fine pubescence	7
–	Pronotum glabrous	8
7(6)	Pronotum much longer than wide (at least 1.5? width) with a narrow strip at middle that is devoid of pubescence; microsculptured and rugulose, but lacking asperites. Cuba	*Caribbomerus exiguus* (Fig. 5g)
–	Pronotum only little longer than wide (about 1.1? width) without a narrow strip at middle that is devoid of pubescence; not microsculptured or rugulose, but with distinct asperites. Puerto Rico	*Caribbomerus charynae* (Fig. 5d)
8(6)	Elytra with matte integument; with micropunctation between punctures	9
–	Elytra with glossy integument; without micropunctation between punctures. Cuba, Puerto Rico, Bahamas	*Caribbomerus attenuatus* (Fig. 5c)
9(8)	Light reddish-brown in color with elytra distinctly paler than pronotum; elytral apices distinctly impunctate; antennal tubercles not elevated, without noticeable depression between. Antigua, Barbuda, Dominica	*Caribbomerus similis* (Fig. 5j)
–	Darker reddish-brown in color with elytra usually no lighter than pronotum; elytral apices not distinctly impunctate; antennal tubercles elevated with distinct depression between them. Hispaniola	*Caribbomerus asperatus* (Fig. 5b)

## Supplementary Material

XML Treatment for Caribbomerus elongatus

XML Treatment for Caribbomerus decoratus

XML Treatment for Caribbomerus asperatus

XML Treatment for Caribbomerus similis
